# Tackling Complex Analytical Tasks: An ISO/TS-Based Validation Approach for Hydrodynamic Chromatography Single Particle Inductively Coupled Plasma Mass Spectrometry

**DOI:** 10.3390/ma13061447

**Published:** 2020-03-22

**Authors:** Yves U. Hachenberger, Daniel Rosenkranz, Fabian L. Kriegel, Benjamin Krause, René Matschaß, Philipp Reichardt, Jutta Tentschert, Peter Laux, Norbert Jakubowski, Ulrich Panne, Andreas Luch

**Affiliations:** 1 Department of Chemical & Product Safety, German Federal Institute for Risk Assessment (BfR), Max-Dohrn-Strasse 8-10, 10589 Berlin, Germany; Yves.Hachenberger@bfr.bund.de (Y.U.H.); Fabian.Kriegel@bfr.bund.de (F.L.K.); Benjamin-Christoph.Krause@bfr.bund.de (B.K.); rene.matschass@gmx.de (R.M.); Philipp.Reichardt@bfr.bund.de (P.R.); Jutta.Tentschert@bfr.bund.de (J.T.); Peter.Laux@bfr.bund.de (P.L.); Andreas.Luch@bfr.bund.de (A.L.); 2 Federal Institute for Materials Research and Testing (BAM), Richard-Willstätter-Strasse 11, 12489 Berlin, Germany; ulrich.panne@bam.de; 3 SPETEC GmbH, Berghamer Str. 2, 85435 Erding, Germany; norbert.jakubowski@spetec.de

**Keywords:** single particle ICP-MS, nanoparticle characterization, nano-carrier, liposomes, hydrodynamic chromatography (HDC), validation

## Abstract

Nano-carrier systems such as liposomes have promising biomedical applications. Nevertheless, characterization of these complex samples is a challenging analytical task. In this study a coupled hydrodynamic chromatography-single particle-inductively coupled plasma mass spectrometry (HDC-spICP-MS) approach was validated based on the technical specification (TS) 19590:2017 of the international organization for standardization (ISO). The TS has been adapted to the hyphenated setup. The quality criteria (QC), e.g., linearity of the calibration, transport efficiency, were investigated. Furthermore, a cross calibration of the particle size was performed with values from dynamic light scattering (DLS) and transmission electron microscopy (TEM). Due to an additional Y-piece, an online-calibration routine was implemented. This approach allows the calibration of the ICP-MS during the dead time of the chromatography run, to reduce the required time and enhance the robustness of the results. The optimized method was tested with different gold nanoparticle (Au-NP) mixtures to investigate the characterization properties of HDC separations for samples with increasing complexity. Additionally, the technique was successfully applied to simultaneously determine both the hydrodynamic radius and the Au-NP content in liposomes. With the established hyphenated setup, it was possible to distinguish between different subpopulations with various NP loads and different hydrodynamic diameters inside the liposome carriers.

## 1. Introduction

Nanoparticles (NPs) have unique physico-chemical properties which makes their application attractive in a broad range of industrial applications [1,2]. Over the recent years an increasing trend of consumer products which contain NPs became evident. They are used for example in cosmetics as energizer in skin creams (gold (Au)-NP), antibacterial reagents (silver (Ag)-NP), abrasives in toothpaste (aluminum oxide (Al_2_O_3_)-NP) and as ultra violet protecting agent (titanium oxide (TiO_2_)-NP). Although NPs are widely used, they exhibit a potential risk to human health by possible release from products or by inhalative and oral uptake by humans [3,4].

The potential hazard of NPs depends on their intrinsic physico-chemical properties (composition, size, concentration and shape), their surface modifications and their interaction with the environment [5,6,7,8,9,10,11]. The latter results in the formation of a biocorona created by complex matrices (e.g., lipids in cosmetics, proteins in cell culture media or bio-fluids) and influence the uptake, dissolution or aggregation behavior of NPs in biological systems [12]. Liposomes are promising carrier systems for the targeted administration of substances in biomedical applications. They are used to deliver drugs such as small interfering ribonucleic acids, to prevent NPs degradation and to target specific cell types [13,14,15]. Additionally, the directed combination of organic and inorganic particulate systems has several advantages. For example, uptake can be controlled and facilitated across different barriers, which is essential for a targeted therapy. With the encapsulation of NPs, the properties of the enclosed particles can be influenced at the nanoscale level [16]. The determination of particle size, shape and composition is vital for the prognosis of biodistribution and the in vivo fate [17,18,19]. Unfortunately, the most commonly used methods (electron microscopy, dynamic light scattering) to characterize NP parameters are strongly influenced by proteins or salts typically found in biological environments [7]. Real samples usually contain low concentrations of various NPs in combination with a high concentration of matrix. Traditionally NPs are extracted from the matrix resulting in a sample that is suitable for the following instrumental analytical techniques. This process can lead to matrix-based artifacts such as NP aggregation and/or even loss. Therefore, a reliable and robust methodology encompassing sample preparation and analysis steps is required, where the physico-chemical properties of the particles are not altered and the measurement in a matrix-containing environment such as liposomes is feasible [20]. 

A possible solution to reduce the influence and content of the matrix is the application of an additional separation step, using a hyphenated approach. Depending on the properties and capabilities of the separation technique, it is possible to separate or concentrate the analytes of interest. Furthermore, the matrix is diluted or exchanged, which typically simplifies NP characterization [21].

The hydrodynamic chromatography (HDC) is a technique, which is able to separate colloidal suspensions through a solid phase of spherical particles. The colloidal suspension is separated based on the size of the colloid, the packaging of the solid phase and the ionic strength of the carrier [22]. The possibility of coupling HDC with other techniques results in an increased use for the characterization of polymers and particle mixtures [23]. Tiede et al. have demonstrated the online coupling of HDC for the pre-separation of NP fractions, and used inductively coupled plasma mass spectrometry (ICP-MS) as a suitable technique to detect a broad range of engineered NPs in complex environmental matrices in a size range of 5–300 nm for standard HDC columns [24,25]. The specific properties of HDC in combination with asymmetric flow-field-flow fractionation (AF4) columns were characterized [26]. This setup includes the possibility to detect metal ions as well as NPs at a low detection limit of 5 µg L^−1^ and runtime below 10 min. With online coupling of HDC or AF4 and spICP-MS Pergantis et al. were able to calculate the particle size and number-based concentrations of low amounts of Au- and Ag-NPs in environmental matrices [27,28]. The hyphenated technique applied in this study, HDC plus spICP-MS, allows to determine the hydrodynamic diameter (r_h_) of a particle and the metallic core diameter of the particle at the same time. The particle core diameter, which is obtained with the spICP-MS is comparable to results based on electron microscopy and is dependent on the particle composition. The particle core diameter can also be used to quantify the constituents of the NPs [23,29,30]. The hydrodynamic diameter is established by HDC separation and is dependent on the environment surrounding the particle and its surface characteristics.

This study applied the guidelines of a conventional spICP-MS and expanded it with an optimized workflow for a coupled HDC-spICP-MS approach to determine NP characteristics with and without liposomes. This procedure was based on a technical specification (TS), which was approved by the International Organization for Standardization (ISO), the European Committee for Standardization (CEN) and the German Institute for Standardization (DIN). The guideline is the CEN ISO/TS 19590:2019 (DIN SPEC 19286:2019-11) [31,32]. Special consideration was paid to reduce measurement time and enhance robustness through an online calibration. The multidimensional capabilities of the coupled techniques could be successful demonstrated for Au-NP-loaded liposomes. Especially promising is the combined separation of liposomes based on their hydrodynamic size in combination with the quantification of their inorganic NP content. 

## 2. Materials and Methods 

### 2.1. Chemicals and Materials

Standard solutions of dissolved Au (1000 mg L^−1^, TraceCERT^®^, Sigma-Aldrich, Darmstadt, Germany), 3.5% nitric acid (technical graded 70% v/v, VWR, Darmstadt, Germany), purified in a douPur quartz sub-boiling point apparatus (MLS GmbH), and Milli-Q water (MilliPore gradient, Merck MilliPore, Darmstadt, Germany) were used for the calibration solutions (0.5, 1, 2, 5 and 10 µg L^−1^). Reference materials of citrate-stabilized Au-NPs (National Institute of Standards and Technology (NIST) 8012 and 8013, Gaithersburg, MD, USA) at a nominal size of 30 and 60 nm were used. Furthermore, Au-NPs in the size of 20, 80 and 100 nm, respectively (NanoComposix, San Diego, CA, USA) were measured. Two cationic liposomes (EL-01-C and EL-11-C, NOF Corporation, Tokyo, Japan) and a neutral liposome (El-01-PN, NOF Corporation, Tokyo, Japan) were used, which contain the following lipid components (µmol/vial): dipalmitoylphosphatidylcholine (DPPC):Cholesterol:Stearyl (52:40:8, EL-01-C in the size of 100–300 nm), 1-palmitoyl-2-oleoyl-sn-glycero-3-phosphocholine (POPC):Cholesterol:Stearyl (52:40:8, EL-11-C in the size of 50–250 nm) and 1,2-distearoyl-sn-glycero-3-phosphoethanolamine-polyglycerine (DSPE-PG8G):DPPC:Cholesterol:1,2-dipalmitoyl-sn-glycero-3-phosphorylglycerol (DPPG) (4.2:20.5:15.2:2.3, EL-01-PN in the size of 50–250 nm). The liposomes were dispersed in Milli-Q water (see above). The reference NP materials NIST 8012 and 8013 were diluted to final concentrations of 85 ng L^−1^ (NIST 8012), 50 and 500 ng L^−1^ (NIST 8013), respectively. The final concentrations used for the 20, 80 and 100 nm Au-NPs were 20, 1000 and 1450 ng L^−1^ respectively. The liposome NP solutions were prepared by using 2.6 g L^−1^ of freeze-dried liposomes and 500 ng L^−1^ of the 30 nm Au-NPs (NIST 8012). After mixing of both constituents, the solution was shaken and vortexed for 10 s. All solutions injected to the HDC column are diluted in an eluent consisting of 0.5% Tween-20 (AppliChem, Darmstadt, Germany), 0.13% sodium dodecyl sulfate (Merck, Darmstadt, Germany) and 5 mM ammonium acetate (Merck, Darmstadt, Germany).

### 2.2. Instrumentation

An HDC column (polymer labs particle size distribution analyser (PL-PSDA) Cartridge Type 1, Agilent, Waldbronn, Germany) with a separation capability of NPs in the size range of 1.5–300 nm are coupled to a volume splitter (with an adjusted ratio of 1:10) via a Y-Piece to an ICP-MS instrument (X-Series, Thermo Scientific, Bremen, Germany). The eluent consisted of ultra-pure water with 0.5% Tween-20 (AppliChem, Darmstadt, Germany), 0.13% sodium dodecyl sulfate (Merck, Darmstadt, Germany) and 5 mM ammonium acetate (Merck, Darmstadt, Germany). The sample injection with a sample loop volume of 0.15 mL was done with a high-performance liquid chromatography (HPLC) autosampler and pump system (Accela 600, Thermo Scientific, Bremen, Germany). The second line port of the Y-piece was connected to a peristaltic pump. The flow rates before and after volume splitting were 1.5 and 0.15 mL min^−1^, respectively. The overall measurement time was 60 s for the conventional spICP-MS and 600 s for the HDC-spICP-MS, followed by a 200 s elution phase. Both experiments used a dwell time of 10 ms, a radio frequency power of 1400 W and a nebulizer argon gas flow rate of 14 L min^−1^. For the conventional spICP-MS setup a PC^3^ Peltier cooler with a cyclonic spray chamber (Elemental Scientific, Mainz, Germany) were used. In order to measure the same number of particulate events with the HDC-spICP-MS, the number concentration of NP solutions was increased by a factor of 10 as described above.

The analysis of the hydrodynamic particle diameters was done either by dynamic light scattering (DLS) (Zetasizer, Malvern Panalytics, Kassel, Germany) or by particle tracking (NTA) (Nanosight LM20, Malvern Panalytics, Kassel, Germany).

### 2.3. Performance and Quality Criteria for spICP-MS

CEN ISO/TS19590:2019 defines several performance and quality criteria (QC) for spICP-MS. Key parameters are namely the transport efficiency, which should be ≥1%, which is calculated as described (e.g., by Degueldre et al.), and the linearity of an external ionic calibration, which is requested to reach a correlation coefficient (R²) of ≥ 0.99 [29]. Moreover, the particle blank solutions should not contain more than 10 particulate events and the overall number of events should be between 100 and a maximum of 10% of the theoretical possible number of events. Furthermore, a general procedure of the measurement (injection list) is outlined, which includes regularly blank (every 10 samples), ionic and particle recovery samples (every 10 samples), along with full calibration samples (every 50 samples). Additionally, equations for the calculation of the limits of detection are given. Nevertheless, the technical specification (TS) is a basic tool, which does not specify an approach for complex samples, coupled setups, or alternative advanced sample introduction systems in general.

### 2.4. Data Analysis Procedure

The raw ICP-MS data were exported as csv-type files and further processed with Microsoft Excel (2016) or Origin Pro 9.1 OriginLab, North Hamption, NH, USA). All calculations were done according to CEN ISO/TS 19590:2019. The used formulas are given in the Supplementary Materials Section 1. The size separated ICP-MS data were binned according to Pergantis et al. with binning sizes of 5 s for the retention time and 2.5 nm for the ICP-MS core diameter [27].

## 3. Results and Discussion

### 3.1. HDC-spICP-MS Setup and Calibration Approach

In this study the QC of the TS of a conventional spICP-MS setup were compared with the results from a coupled HDC-spICP-MS system. The applicability of the QC, along with the proposed additional QC for the evaluation of a coupled system, was evaluated. The application of a hyphenated separation technique based on spICP-MS can be time-consuming due to the separation technique-dependent dead time, the separation process and essential calibrations. However, after a proper installation and optimization, such a setup allows to generate additional information contributing to an improved particle characterization that results in a multidimensional size determination.

The TS suggests determining the QC, based on particle standards every 10 samples (excluding blank measurements) and ionic calibration every 50 samples. However, due to the increased measurement time per sample with a coupled system a drift of the instrumental parameters is likely to occur, especially in the case of intensity values. To prevent the infringement of the QC, which would result in incorrect quantification, an online calibration approach was developed. This approach enables to determine the QC for all runs of the whole sample sequence and the calculation of the drift. 

**Figure 1:**
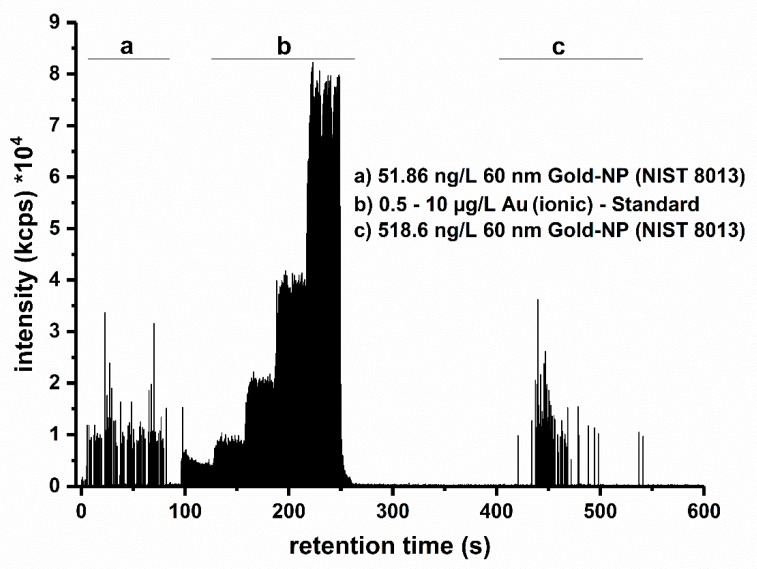
Exemplary HDC-spICP-MS measurement of Au NP (NIST 8013, 60 nm) with the online calibration approach. This includes the measurement of a reference particle for the determination of the transport efficiency (section a, number of spikes: 118, transport efficiency: 4.4%), and a five-point ionic calibration (section b, sensitivity: 5699.12 cps (ng mL^−1^)^−1^). Both preparations are being directly injected through a Y-Piece located between the HDC and ICP-MS instrument. The HDC-separated 60 nm Au-NPs were detected in section c (number of spikes: 173, average signal intensity: 9142 counts).

For the hyphenation of HDC a Y piece was placed behind the column to inject standards into the eluent for an online calibration. This approach allows the injection of all necessary standards via a peristaltic pump during the dead time gap of the system (see Figure 1). The calibration procedure was comprised of two steps. At first the transport efficiency of the ICP-MS system was determined with an NP reference solution (around 50 ng L^−1^ of 60 nm Au-NPs NIST 8013). Calibration solutions of 0.5, 1.0, 2.0, 5.0 and 10 µg L^−1^ were injected for 30 s each. The averaged signal intensities were used to calculate the sensitivity with a linear regression approach. This was followed by a sensitivity check of the analyte, achieved with the five-point ionic calibration linearity assessment. At the end of each run only separated NPs are being detected. 

### 3.2. Determination of Quality Criteria Based on the Technical Specification (TS)

As a first step both setups—conventional spICP-MS and coupled HDC-spICP-MS—were characterized based on their spICP-MS capabilities. The QC parameters used in this study were taken from the ISO/TS 19590:2017 and adopted to convert the general approach of the TS towards hyphenated methods using spICP-MS. 

The experiments were performed using analyte blank samples as well as ionic solutions of Au in the range of 0.5 to 10 ng mL^−1^ (n = 3). The measured intensities of these samples were used to determine the analyte sensitivity, linearity over the investigated concentration range and to estimate the limit of detection (LOD) in mass as well as to predict the LOD for the particle size. Both setups were working within the defined QC range (see Table 1). Comparison of the conventional spICP-MS setup with the coupled approach shows a small increase of the sensitivity (3879.44 to 5675.94 cps (ng mL^-1^)^-1^), which results in a lower LOD in mass of the ionic concentrations (0.028 to 0.021 ng mL^−1^). The predicted LOD in particle size (12.7 nm spICP-MS and 10.3 nm HDC-ICP-MS) is in agreement with values from literature [33]. All LOD values related to particle size were calculated with an iterative approach (see supporting information).

The linearities of the calibration curves for both setups match the TS criteria (R² > 0.99). A slightly lower R² value was obtained for the HDC-spICP-MS setup, which can be attributed to instrumental differences (e.g., the injection of the ionic solutions by using the Y-piece). QC were comparable for both systems showing the applicability of the chosen online calibration procedure. 

Additional requirements of the TS are the following: a specific injection list, which includes regularly blank solutions and calibration samples. This is applied in the experimental design and not further discussed here. Lastly, a limit of particulate leftovers in the blank solution, as well as a range of particulate events between 100% and 10% of the theoretical detectable particles is defined. The particulate leftovers of the HDC-spICP-MS setup were counted with a mean of 6 ± 3 NPs per blank sample. The number of particulate events was around 99 ± 8 NPs for the conventional spICP-MS with a volume of 0.15 mL and a flow rate of 0.15 mL min^−1^. HDC-spICP-MS achieved 183 ± 15 NPs particulate events in an injected sample volume of 0.1 mL with roughly the same flow rate due to a 1:10 variable flow splitter. To compensate for the flow splitter, the concentration used with the HDC-spICP-MS setup was increased by a factor of 10. The additional separation step led to an improved NP transportation and thus an increasing particle number detected. A direct comparison of both data sets is shown in the supplement Table S1. Our results indicate a proper fulfillment of the QC to further advance and measure NPs in solution with HDC-spICP-MS setup.

### 3.3. Determination of Quality Criteria (QC) for Nanoparticle Solutions Based on the Technical Specification (TS)

As a second step, NP solutions (60 nm; 8013 NIST; 500 pg mL^−1^ and 50 pg mL^−1^) were analyzed with and without the coupled HDC (see Table 2) in 5 repeats at one day to test the repeatability. Additionally, day-to-day variations of the HDC as well as of the spICP-MS may heavily affect the measurement quality. Therefore, it was decided to determine the QC parameters on a daily basis as they are paramount for many essential parameters. For example, they are also relevant for NP quantification, namely the number of particles, the calculated transport efficiency, and they affect the determination of the regular blank and calibration solution.

For the evaluation of the transport efficiency two different approaches are mentioned in the TS, the counting method and the size method. The correct selection of the appropriate calculation approach for the transport efficiency is dependent on the availability of a NP reference material. If a certified NP size standard with a known particle number concentration is used the so-called counting method can be applied. When the particle number concentration of the certified NP standard is not available the size method should be used, in which an ionic calibration serves as a substitute [34]. 

Both methods were employed to determine the transport efficiency, and no significant differences were found (counting method: 2.7 ± 0.2%, size method: 2.5 ± 0.2%). Since a certified NP standard with a known particle number concentration was available the counting method was used for subsequent data evaluation.

In order to evaluate the effects of a change from the spICP-MS to the HDC-spICP-MS setup the same Au-NP (NIST 8013) was measured. The resulting particle size of 56.8 ± 1.5 nm for spICP-MS and 56.2 ± 1.1 nm for HDC-spICP-MS shows only a minor deviation and the detected size agrees with the expected values of 56.0 ± 0.5 nm from TEM data provided in the manufacturers certification sheets. It was noted, that the transport efficiency increases from 2.54% for spICP-MS to 6.86% for HDC-spICP-MS (calculated on predicted concentration after the splitter) for the 60 nm Au-NP. The increase can be mainly attributed to the additional use of the pre-separation HDC step which results in a lower frequency of particles reaching the nebulizer and spray chamber. Although differences in the transport efficiencies of the two setups are determined, the conditions of the QC (above 1%) are fulfilled. Therefore, the QC of the TS is reached with our methods. 

### 3.4. Additional Validation for Multidimensional Coupled Techniques

Nevertheless, it is beneficial to also account for the additional capabilities of the coupled setup, which are not considered by the QC of the TS. Therefore, we propose here an alternative transport efficiency calculation, a cross calibration between the spICP-MS and the HDC-spICP-MS as well as the determination of the resolution of the separation. 

#### 3.4.1. Modified Transport Efficiency

As described above, the calculation of the particle size relies on the determined transport efficiency of the spICP-MS setup. Due to the hyphenated setup additional considerations have to be taken into account to guarantee trust- and meaningful robust results. Therefore, different NP sizes were tested to confirm the independence between transport efficiency (η) and NP size.

**Figure 2:**
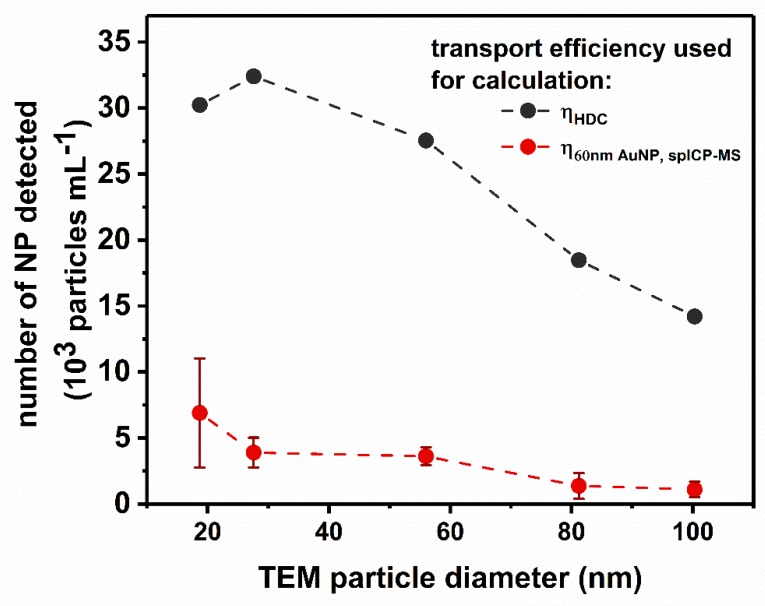
Au-NP number concentrations for different sizes as obtained by HDC-spICP-MS considering the size-dependent transport efficiency: (1) according to the TS (red dots, η_60nm AuNP_), (2) size-dependent (black dots, η_HD_).

In case of a coupled setup, the experimental setup of the HDC column, the flow splitter, connection pieces and additional tubing have to be taken into account, when considering particle loss and size-dependent transport efficiencies. To investigate this issue and also the robustness of the coupled HDC-spICP-MS system, five different Au-NPs (i.e., 20, 30, 60, 80 and 100 nm) in comparable number concentrations (20, 85, 516, 1000, 1450 ng mL^−1^) were measured 6 times during the period of one month. For the HDC, a size-depended loss mechanism can be observed (see Figure 2). The effect is increasing from smaller to bigger particles (η_HDC_ see Table 3). The particle number concentration was determined as described before (using the 60 nm Au-NP NIST 8013, measured by spICP-MS). The obtained values reveal a strong underestimation (77% to 94% for 20 to 100 nm) as shown in Figure 2 (red spots). However, applying the size-dependent transport efficiency this falsification can be corrected. Under these conditions, the expected values are obtained (see Figure 2, black spots). 

#### 3.4.2. Cross Validation of NP Size Determination

For an evaluation of the size determination of the HDC-spICP-MS the particle sizes measured have been compared to the results from TEM and DLS. 

HDC-spICP-MS and TEM methods characterize the core particle diameter. The HDC-spICP-MS values obtained are slightly higher as the expected values obtained by TEM measurements. To define a QC the expected particle sizes (TEM) were plotted against the detected particle sizes by spICP-MS. The acquired linear fit has an accuracy deviation of 4.5% (R² = 0.9921) (see supplement Materials Figure S1). Based on the TS such a correlation coefficient proves the values obtained with spICP-MS in the coupled setup. Those values are in line with the literature (4.8%) [35].

As a next step the capabilities of the HDC-based size determination and separation was evaluated. The Au-NPs of varying sizes (see Table 3) were required to pass through the HDC column, which retains them differently based on their hydrodynamic sizes (1.5–300 nm). Bigger particles elute faster compared to the smaller ones. One approach to calculate the retention time-dependent size was shown by Mitrano et al. and Pergantis et al. [27,28].

In this study a similar approach was chosen to compensate for differences of the hydrodynamic diameter compared to the core size of Au-NPs, DLS measurements were done with different particle sizes (20–100 nm, in HDC eluent (supplement Figure S2)). The obtained values were used for the calibration of the HDC. The obtained hydrodynamic diameters were plotted against the mean retention time of the HDC, with time points over one month. A comparable behavior (accuracy deviation: 5.2%, R² = 0.9853 see Figure S3) as observed above can be seen for both hydrodynamic diameters measured with DLS as the reference technique and the HDC. 

Both cross calibrations can be used as additional QC (accuracy and R²) and are in good agreement with the TS for the determination of particle sizes. In case of the core size determination with HDC-spICP-MS data the same criteria as in the TS (R² > 0.99) is applied. This value is purely based on the instrumental performance, because it only takes the ion or particle response of the ICP-MS into account.

In the case of the hydrodynamic size determination, which is independent of material composition, the result of the cross calibration is affected by the instrumental-dependent factors. Those are more prone to variation for both techniques. Therefore, the criterion was set as R² > 0.98 of the plot as shown in supplement Figure S3. However, the results of the NP core sizes, hydrodynamic sizes and their number concentrations are in good accordance with the complementary techniques. Our findings are in agreement with data provided by literature [35]. 

Based on these findings, the introduced HDC-spICP-MS setup can analyze the inorganic composition (see spICP-MS data before) as well as the hydrodynamic diameter of particles in parallel. Moreover, the results are in line with the QC of the TS and also serve as an example of additional QC for a hyphenated setup (accuracy and R²). The application of different cross calibration techniques generates an increase in accuracy for the respective particle size measurements. Due to this the presented approach is especially useful, if the analytes of interest consists of organic as well as inorganic compounds, which cannot be differentiated by either technique alone.

#### 3.4.3. Separation Capabilities of HDC-spICP-MS

**Figure 3:**
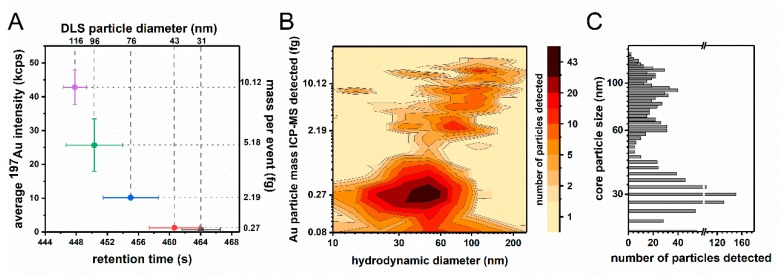
(**A**) Cross calibration of the HDC-spICP-MS with hydrodynamic (DLS) and the particle mass per event based on the core diameters from TEM. (**B**) Spike fractogram of a mixture of 4 differently sized Au-NPs. (**C**) Frequency based distribution of measured core particle sizes.

The next series of experiments were focused on the separation capabilities of the combined HDC-spICP-MS approach. Increasingly complex mixtures of the before used Au-NPs (60/100; 30/60; 60/80; 30/60/100 and 30/60/80/100, see Table 4) were measured to demonstrate the possible separation capacity according to different size subpopulations. An overview of the cross calibration, as described in Section 3.4.2, is shown in Figure 3A. The resolution was calculated as an adaptation of liquid chromatography (see schematically description in Figure S4). Typically, 2 peaks are thought of being resolved if the resolution is above at least 1.5. This value is achieved for most of the particle mixtures, except for the most complex one. A summary of these values is given in Table 4. A representative spike fractogram of the particle mixtures with 4 sizes is shown in Figure 3B. This was achieved with a multiple peak fit function of the Peak Analyzer tool (Origin Pro 9.1). Additionally this procedure may include a background subtraction depending on the calculated peak overlaps. Later, due to the broad size distribution, this background subtraction (particle number-based) was used to differentiate between different size subpopulations. The parameters used are shown in Table 4. With this approach the resolution of a mixture of four particle sizes and their respective maxima was found overall enhanced (18.5/0.4/0.4 to 5.3/2.2/4.0). The uncorrected frequency-based distribution is shown in Figure 3C. 

To sum up, the size determination (hydrodynamic and core diameter) of different Au-NP mixtures with increasing complexity was successfully achieved. Based on these results this method is feasible to detect different subspecies of NPs with size differences of about 20 nm. A QC based on these results for a hyphenated setup is the evaluation of a size mixture of at least 2 known particles. The separation must be evaluated as described before. The satisfying separation is achieved if the resolution is above 1.5.

### 3.5. Proof-of-Concept Application—Au-NPs Embedded in Liposomes

In order to apply our validated HDC-spICP-MS to a relevant complex problem, hydrodynamic sizes and metallic/inorganic content of lipid-based nano-carrier systems were investigated. The HDC-spICP-MS setup should be able to differentiate between similar sized lipids based on their inorganic content. Therefore, an estimation about their particulate content as well as information about the hydrodynamic size of the loaded liposome is required. 

To test the exemplary liposome EL-01-C without particulate content, NTA was conducted (see supplement Figure S5). The NTA derived hydrodynamic size of 182.0 ± 7.8 nm is in rough agreement with the HDC-UV-Vis based values from Helsper et al. of 205 ± 6 nm [36]. They reported a large peak width at half height, which indicates a rather polydisperse sample. The polydispersity was also confirmed in the NTA and explains the deviation of the NTA based size value. 

**Figure 4:**
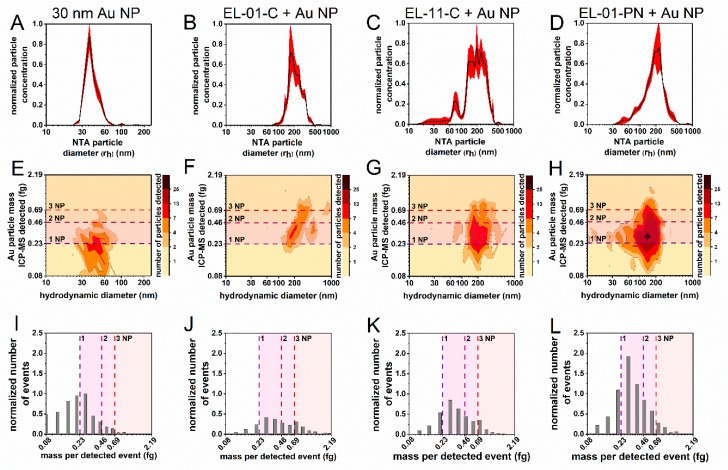
NTA and HDC-spICP-MS results of 30 nm Au-NP with and without the liposomes EL-01-C, EL-11-C and EL-01-PN. The upper panel (**A**–**D**) shows the NTA derived particle size distribution and the middle panel (**E**–**H**) displays the spike fractograms of the HDC-spICP-MS, which are normalized to the maximum of detected particle mass. The lower panel (**I**–**L**) shows the number of particle events per mass detected normalized to the results of the measurement of pure Au-NPs. Dashed lines represent the theoretical mass of one or multiple 30 nm Au-NPs.

The loading of 30 nm Au-NPs leads to a visible size shift in the NTA derived size from 38 nm to around 200 nm for all 3 investigated liposomes (Figure 4A–D). The HDC-spICP-MS results are in good agreement with the NTA data. Furthermore, they show a fraction of liposomes with a hydrodynamic size below 150 nm. These fractions are possibly due to liposome fragments, which are disrupted during the preparation steps or have not formed a properly closed liposome carrier or Au-NPs only (Figure 4F–H). 

The majority of the loaded liposomes contained one NP as indicated by the first dashed line (see Figure 4J–L). Available literature demonstrates the formation of a lipid layer around Au-NPs [37]. However, it remains challenging to localize the Au-NPs within the loaded liposome carrier complex. Modification and disruption of the formation, uptake and rearrangement steps may additionally influence this analytical question. With the HDC-spICP-MS a differentiation between absorbed, partially absorbed and adsorbed Au-NP as part of the loaded liposome carrier is not possible. For biomedical applications TEM measurements are recommended in order to ensure a safe, uniform and reliable carrier system [37]. A comparison of the HDC-spICP-MS data between the dispersed NPs and the loaded liposomes shows a tailing, which indicates the occurrence of multiple particles (up to several NPs based on their mass, Figure 4I–L). The Figure 4I–L display the sum of all Au-NP fractions, which are mentioned before.

The different liposome compositions influence the hydrodynamic size distribution of the liposomes (Figure 4B–D) and their loading with Au-NP (Figure 4F–H) measured with HDC-spICP-MS. Zeta potential measurements of the samples indicate different surface potentials of EL-01-C and EL-11-C (both cationic), and the anionic liposome EL-01-PN (see supplement Table S2). The portion of Au-NPs per liposome seems to be in correlation with the zeta potential (see Figure 4F–H). The more negative the zeta potential of empty liposomes is, the higher is the number of detected Au-NPs during the HDC-spICP-MS measurement (see Figure 4F–H). 

Strong anionic liposomes such as El-01-PN initially repel the negatively charged Au-NP. This hypothesis is supported by a larger fraction of unloaded Au-NP (see Figure 4H). However, absorbed Au-NPs seem to be electrostatically stabilized within the EL-01-PN liposome that may prevent diffusion out of the anionic carrier complex. Liposomes which display a strong cationic behavior such as El-01-C are prone to electrostatic attraction of the Au-NP which may lead to an enhanced adsorption. The shift of zeta potential in the case of the slightly cationic liposome El-11-C after Au-NP loading suggests a combination of both effects.

The hydrodynamic size measured with DLS of empty and loaded liposome carriers indicates an increase (EL-01-C), decrease (EL-11-C) or no size change (EL-01-PN, see supplement Table S2). These responses are in agreement with the different findings of the Au-NP distribution and uptake with different liposome compositions. Therefore, the liposome composition may influence the uptake mechanism of the Au-NPs as well as the stabilization of the loaded liposome carrier complex. Nevertheless, further research has to be conducted to elucidate the mode of action.

The HDC-spICP-MS methodology allows a multidimensional evaluation of Au-NPs in loaded liposomes while still fulfilling all QC. This hyphenation approach provides a rapid analysis of these model compounds.

## 4. Conclusions

In this study a tiered approach for the validation of a hyphenated HDC-spICP-MS setup was investigated. Firstly, an integrated online-calibration routine was established, to reduce the required measurement time as well as to enhance the robustness of the coupled method (see Figure 1). The QC for the conventional spICP-MS of the TS were verified and compared with and without the coupled HDC. Criteria were namely the linearity of the ionic calibration, the transport efficiency of an NP reference standard, a minimum number of particulate events per measurement and the avoidance of particulate leftovers (memories), as well as a standardized sample list, which integrates blank and calibration samples in defined frequencies. Furthermore, additional QC were defined for hyphenated spICP-MS methods. This includes a cross calibration of the determined hydrodynamic and core sizes with additional comparable techniques. The linearity of the resulting plots may be used as QC. To compensate the influences of the coupled technique on the detected NP number, a modified size-dependent transport efficiency was used. With these modifications it is possible to characterize the capabilities of hyphenated method.

The validated HDC-spICP-MS was applied for a complex sample system. For this purpose, the hydrodynamic sizes and metallic/inorganic content of lipid-based nano-carrier systems were investigated. It could be shown that the HDC-spICP-MS setup was able to differentiate between similar sized lipids. Additionally, the inorganic content could be evaluated. The particle uptake distribution of the loaded liposomes can be obtained in parallel with the actual hydrodynamic size of these nano-carrier systems. This distinction between loaded and free NPs is not possible with the conventional spICP-MS.

## Figures and Tables

**Table 1:** Quality criteria parameters of ionic calibration solutions.

Ionic Measurements	spICP-MS	HDC-spICP-MS
Sensitivity (cps (ng mL^−1^)^−1^)	3879.44	5675.94
R^2^	0.99996	0.99587 (0.00050)
LOD (ng mL^−1^)	0.028 (0.001)	0.021 (0.001)
LOD (nm)	12.7 (0.1)	10.3 (0.2)

**Table 2:** Repeatability test with 5 measurement of 60 nm Au-NPs (Au NIST 8013) in one day using spICP-MS or HDC-spICP-MS.

Particulate Parameters	spICP-MS	HDC-spICP-MS
Size (nm)	56.8 (1.5)	55.2 (1.1)
Number of particles detected (×10³ mL^−1^)	26.7 (2.3)	26.5 (2.2)
Transport efficiency (%) of counting method	2.54 (0.19)	6.86 (0.56)
Recovery (%)	100 (± 9)	100 (± 8)

**Table 3:** Recalculated average NP sizes based on different NP solutions in (nm) (n = 6, at 6 days).

TEM ^†^	spICP-MS	DLS	HDC	η_HDC_ (%)
18.7	22.6 (0.8)	31.2 (0.5)	34.4 (5.7)	1.12 (0.34)
27.6	31.6 (1.1)	43.0 (0.5)	39.3 (2.3)	0.61 (0.18)
56	58.2 (1.0)	76.3 (1.1)	73.9 (19.1)	0.50 (0.30)
81.2	83.4 (8.1)	96 (0.8)	103.6 (24.7)	0.45 (0.27)
100.3	104.5(14.0)	115.9 (0.7)	119.5 (12.2)	0.42 (0.17)

**^†^**. TEM values are given from manufactures certification sheets.

**Table 4:** Peak resolution for particle size mixtures without and with background correction.

Separated Size (nm)	Peak Resolution (Number Distribution)	Background (Number of NP)	Background Corrected Peak Resolution (Number Distribution)
30/60	2.12	1.5	3.14
60/80	1.82	2.4	1.49
60/100	1.86	3.3	5.16
30/60/100	1.86/1.54	1.5	3.06/2.05
30/60/80/100	18.51/0.36/0.38	11.5	5.3/2.22/4.03

## Supplementary Materials

The following are available online at https://www.mdpi.com/1996-1944/13/6/1447/s1, Equations (S1)–(S4): Calculation of the instrumental transport efficiency; Equations (S5) and (S6): Estimation of the particle size; Equations (S7)–(S9): Estimation of the particle number concentration; Equation (S10): Estimation of the background; Table S1: Comparison of spICP-MS and HDC-spICP-MS measurements of blank solutions and 60 nm Au-NPs (Au NIST 8013, 50 ppt) over 1 month (n = 5, at 5 days); Table S2: DLS measurements of the hydrodynamic diameter (d_H_) and zeta potential for the used 30 nm Au-NP, lipids only and the lipids loaded with 30 nm Au-NP (n = 3); Figure S1: Expected (TEM) versus quantified average NP size measured with ICP-MS; Figure S2: (A) DLS-derived hydrodynamic size of different Au NPs (20–100 nm) in the eluent of the HDC (n = 6). (B) Calibration curve for HDC retention time with DLS measured hydrodynamic diameters; Figure S3: Expected (DLS) versus quantified average NP size calculated by using the HDC retention time; Figure S4: Schematically description for the estimation of resolution (R) between 2 different size populations (30 and 60 nm Au-NP) in accordance to LC-MS techniques; Figure S5: NTA derived particle number distribution for unloaded liposome EL-01-C.
