# Supplementary material for: Tackling Complex Analytical Tasks: An ISO/TS-Based Validation Approach for Hydrodynamic Chromatography Single Particle Inductively Coupled Plasma Mass Spectrometry
Source: Materials (Basel). 2020 Mar 22;13(6):1447. doi: 10.3390/ma13061447 (PMC7143856; doi:10.3390/ma13061447)
Supplement: Supplementary file 1 [file materials-13-01447-s001.pdf]

materials

MDPI

Supplementary Materials

Tackling Complex Analytical Tasks: An ISO/TS-Based Validation Approach for Hydrodynamic Chromatography Single Particle Inductively Coupled Plasma Mass Spectrometry

Yves U. Hachenberger [1,†] and Daniel Rosenkranz [1,*,†], Fabian L. Kriegel [1], Benjamin Krause [1], René Matschaß [1,2], Philipp Reichardt [1], Jutta Tentschert [1], Peter Laux [1], Norbert Jakubowski [3], Ulrich Panne [2] and Andreas Luch [1]

[1] Department of Chemical & Product Safety, German Federal Institute for Risk Assessment (BfR), Max-Dohrn-Strasse 8-10, 10589 Berlin, Germany
[2] Federal Institute for Materials Research and Testing (BAM), Richard-Willstätter-Strasse 11, 12489 Berlin, Germany
[3] SPETEC GmbH, Berghamer Str. 2, 85435 Erding, Germany
[*] Correspondence: Daniel.Rosenkranz@bfr.bund.de
[†] These authors contributed equally.

1. Calculation of the Instrumental Transport Efficiency

Our study employed two approaches for determination of the instrumental transport efficiency.

1.1. The Counting Method

The transport efficiency $\eta_{counting}$ (%) is calculated as:

$$\eta_{counting} = (60 \times q_P)/(c_P \times \dot{\upsilon}) \times 100\%, \quad (S1)$$

with q_P—particle stream into the plasma (particle × s^{-1}), c_P—particle number concentration (particle × mL^{-1}) and $\dot{\upsilon}$—as the sample uptake rate (mL × min^{-1}).

1.2. The Size Method

The transport efficiency of the size method η_{size} (%) is calculated as:

$$\eta_{size} = (R_{ionic}/R_P) \times 100\%, \quad (S2)$$

R_{ionic} is defined as:

$$R_{ionic} = (RF_{ion} \times 6 \times 10^4)/(\dot{\upsilon} \times t_d), \quad (S3)$$

RF_{ion}—analyte sensitivity (cps(ng mL^{-1})$^{-1}$) t_d—dwell time (ms) and

$$R_P = (\bar{I}_P/m_P), \quad (S4)$$

with $\bar{I}_P$—background corrected average particle intensity (cps) and m_P—mass of particle (µg).

2. Estimation of the Particle Size

The particle size d_P (nm) is defined as:

$$d_P = [(6 \times m_P)/\pi \times p_P)]^{-(1/3)} \times 10^4, \quad (S5)$$

p_P corresponds to the bulk material density (g mL^{-1}). The transport efficiency (η), which was derived from either the counting method (see Equation (S1)) or the size method (see Equations (S2)–(S4)is now used to calculate m_P;

$$m_P = [(I_P \times t_d)/(RF_{ion})] \times [(\dot{\upsilon} \times \eta)/60] \times (M_P/M_a), \quad (S6)$$

I_P—background corrected particle intensity (cps), M_P—molar mass of the particles and M_a—the molar mass of the analyte.

3. Estimation of the Particle Number Concentration

The particle number concentration c_P (particles × L^{-1}) is considered as:

$$c_P = (d_P/\eta) \times (1000/\acute{\upsilon}). \quad \text{(S7)}$$

3.1. Limits of Detection for the Particle Number Concentration

The limit of detection for the number based concentration LOD_{NP} (particle × L^{-1}) is calculated as:

$$LOD_{NP} = (\acute{n}_P + 3 \times SD_P)/(\eta \times \dot{\upsilon} \times t_a), \quad \text{(S8)}$$

$\acute{n}_P$—average number of particles in blank samples (particles), SD_P—standard deviation of the average number of particles and t_a—total measurement time of each experiment (min). The limit of detection for the mass based concentration LOD_{MP} (ng × L^{-1}) is defined as:

$$LOD_{MP} = LOD_{NP} \times \bar{m}_P, \quad \text{(S9)}$$

$\bar{m}_P$—average particle mass (ng).

4. Estimation of the Background

Determination of the ionic background intensity (I_{BG}) was in accordance to the 3σ-approach with:

$$I_{BG} = \acute{I}_{BG} + 3 \times I_{SD}, \quad \text{(S10)}$$

$\acute{I}_{BG}$—average intensity of the original background, I_{SD}—standard deviation of the average background intensity.

5. Additional Tables

Table S1. Comparison of spICP-MS and HDC-spICP-MS measurements of blank solutions and 60 nm Au-NPs (Au NIST 8013, 50 ppt) over 1 month (n = 5, at 5 days).

Parameters	spICP-MS	HDC-spICP-MS
Number of particulate events (blanks)	6 (3)	6 (3)
Number of particulate events (samples)	99 (8)	183 (15)
Transport efficiency (%)	2.94	8.74
Number of particles per run (×10³ mL⁻¹)	26.7 (2.1)	26.7 (3.9)
Expected number of particles (×10³ mL⁻¹)	26.7	26.7

Table S2. DLS measurements of the hydrodynamic diameter (d_H) and zeta potential for the used 30 nm Au-NP, lipids only and the lipids loaded with 30 nm Au-NP (n = 3).

-	r_H (nm)		zeta potential (mV)	
**30 nm Au-NP**	**43.05 (0.5)**		**−21.7 (1.2)**	
-	empty	loaded	empty	loaded
El-01-C	204.4 (1.4)	231.1 (4.7)	75.4 (0.7)	37.5 (2.8)
El-11-C	163.9 (1)	149 (1.6)	27.1 (2.1)	-30.3 (0.3)
El-01-PN	175.7 (1.1)	177.1 (0.7)	-76 (1.9)	-87.9 (1.5)

6. Additional Figures

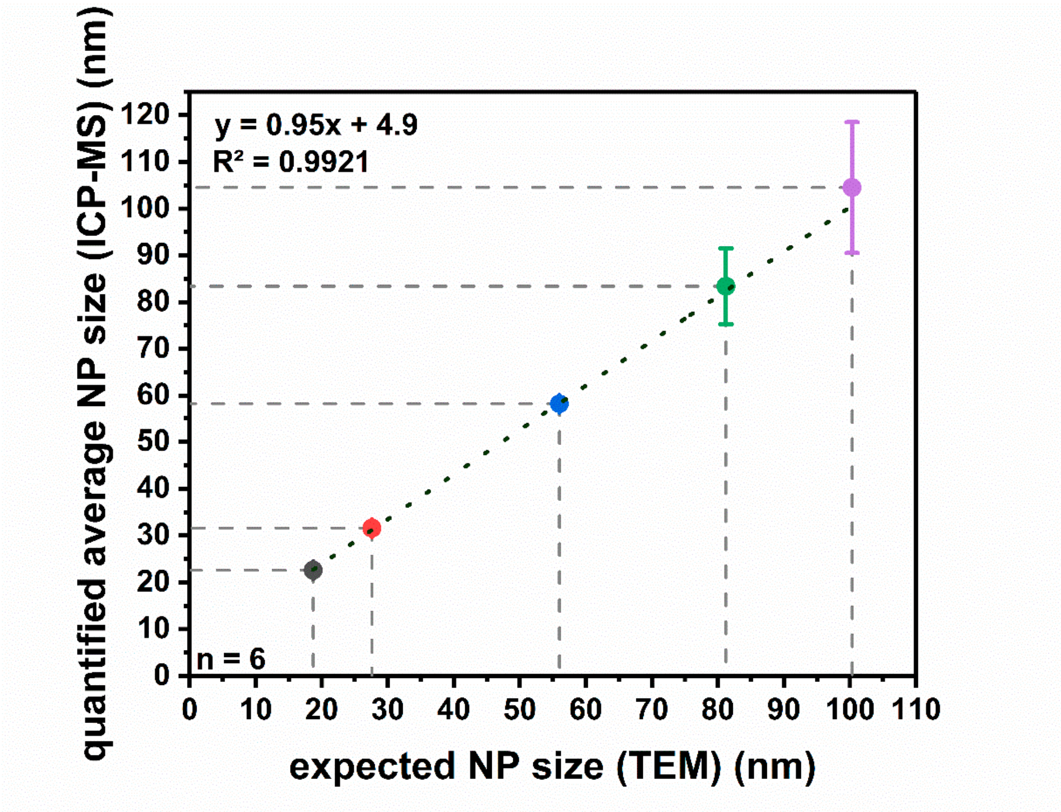

Figure S1. Expected (TEM) versus quantified average NP size measured with ICP-MS.

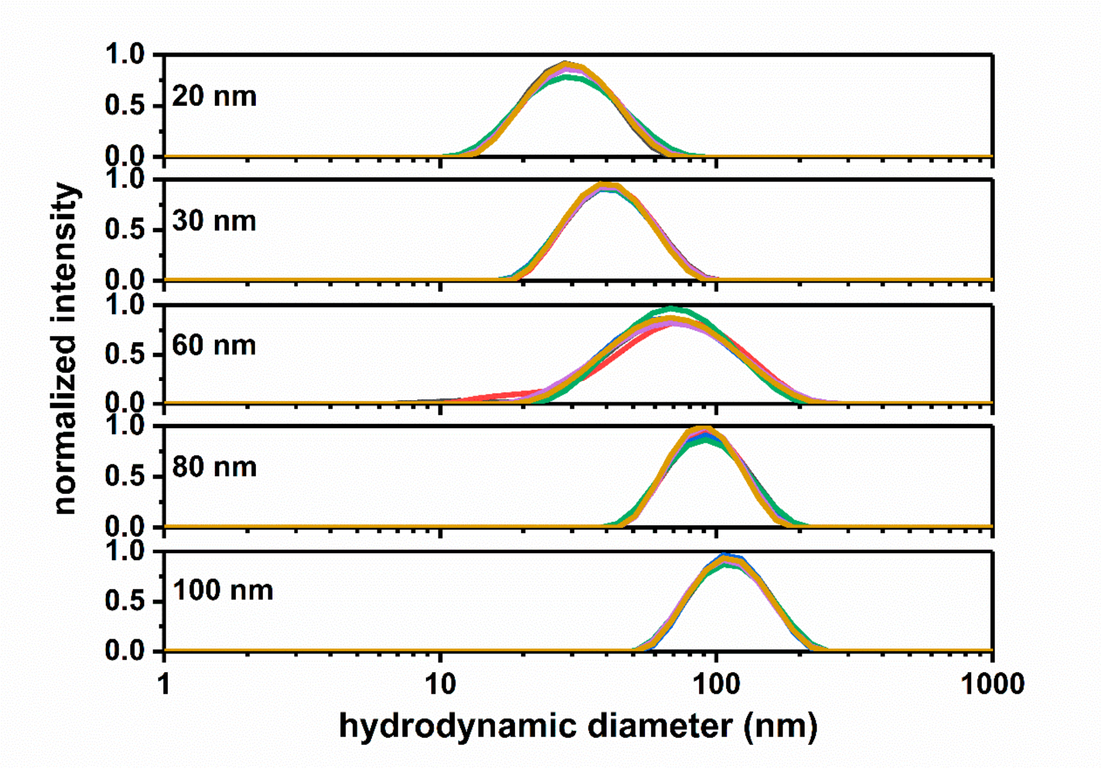

(**A**)

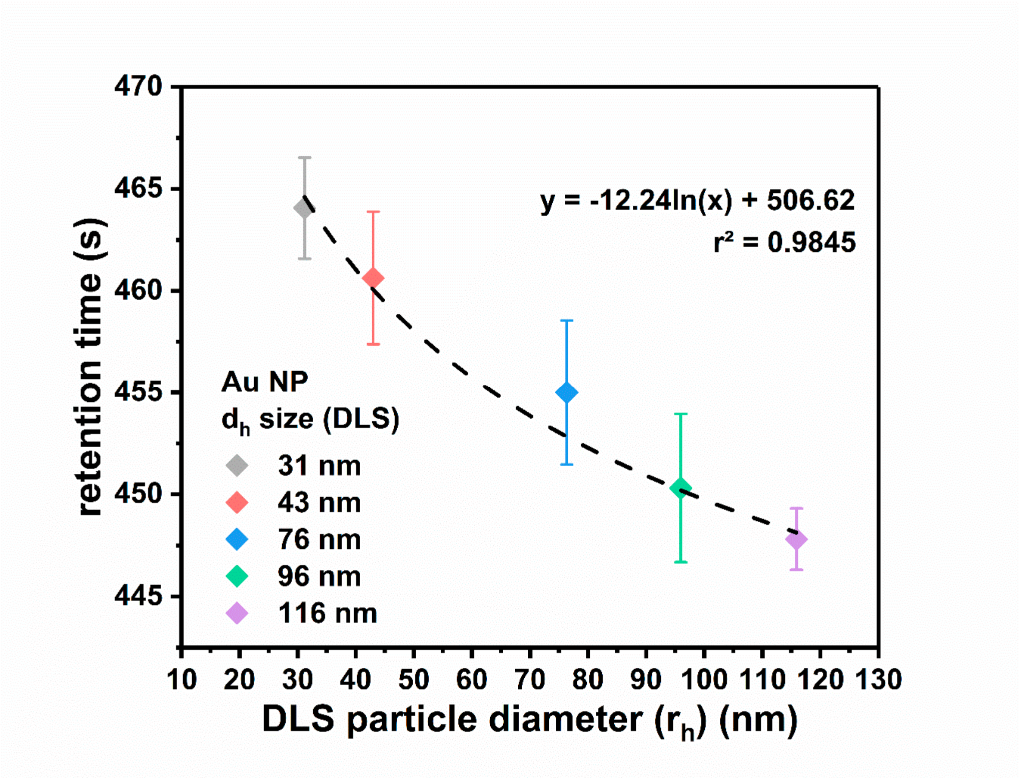

(**B**)

Figure S2. (**A**) DLS-derived hydrodynamic size of different Au-NPs (20–100 nm) in the eluent of the HDC (n = 6). (**B**) Calibration curve for HDC retention time with DLS measured hydrodynamic diameters.

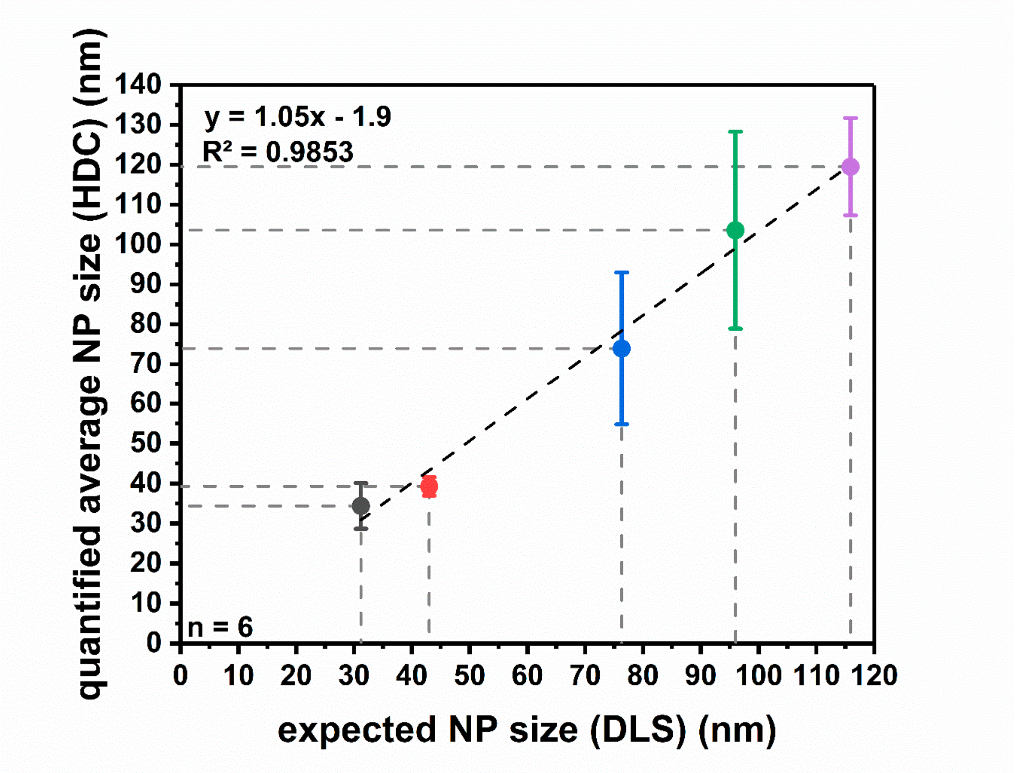

Figure S3. Expected (DLS) versus quantified average NP size calculated by using the HDC retention time. For both techniques the particles are measured in the eluent used for the HDC-spICP-MS.

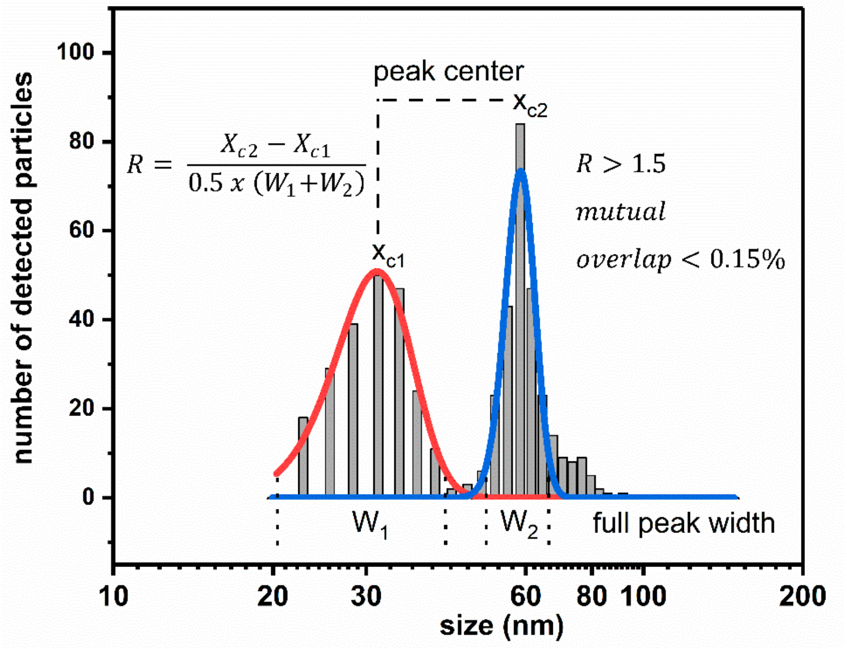

Figure S4. Schematically description for the estimation of resolution (R) between 2 different size populations (30 and 60 nm Au-NP) in accordance to LC-MS techniques.

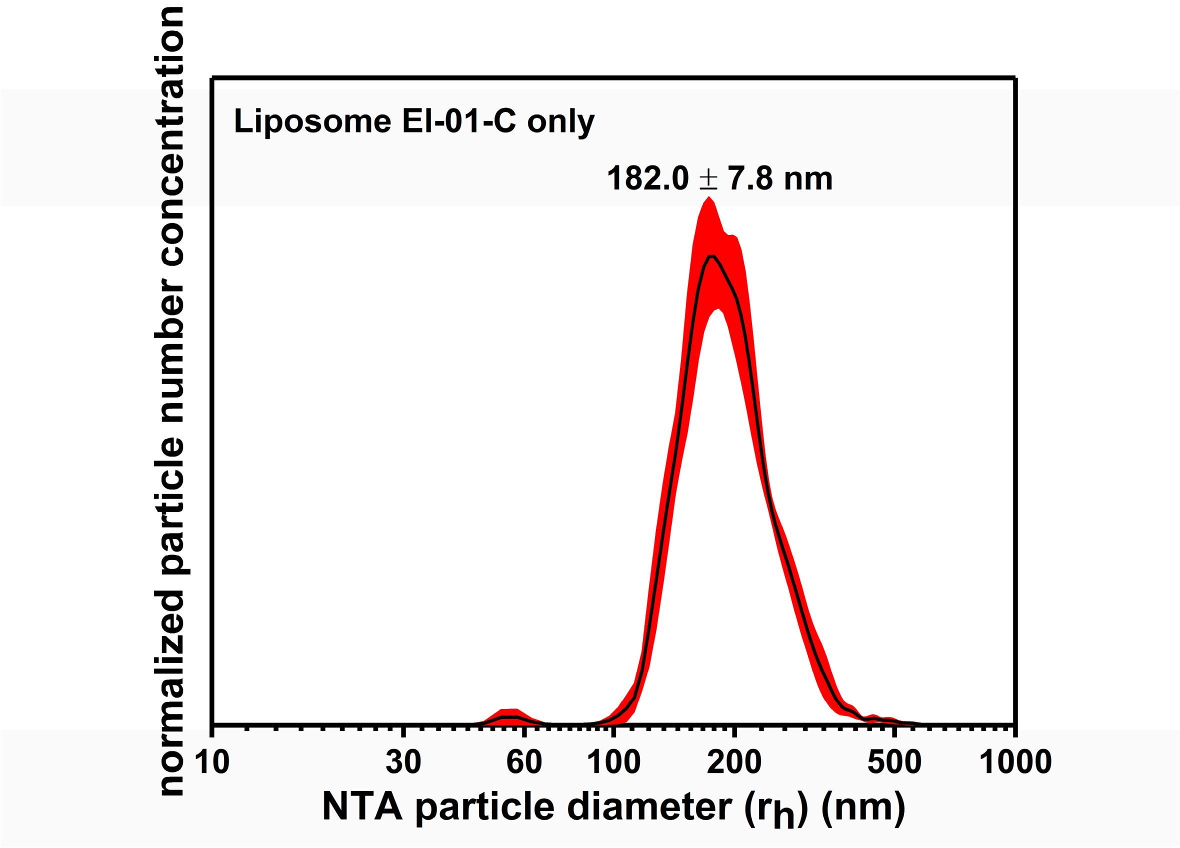

Figure S5. NTA derived particle number distribution for unloaded liposome EL-01-C.

© 2020 by the authors. Licensee MDPI, Basel, Switzerland. This article is an open access article distributed under the terms and conditions of the Creative Commons Attribution (CC BY) license (http://creativecommons.org/licenses/by/4.0/).
